# Security, Privacy, and Trustworthiness of Sensor Networks and Internet of Things

**DOI:** 10.3390/s20143846

**Published:** 2020-07-10

**Authors:** Sokratis Katsikas, Vasileios Gkioulos

**Affiliations:** 1 Department of Information Security and Communication Technology, Norwegian University of Science and Technology, 2815 Gjøvik, Norway; vasileios.gkioulos@ntnu.no; 2 Faculty of Pure and Applied Sciences, Open University of Cyprus, Latsia 2220, Cyprus

**Keywords:** security, privacy, trustworthiness, Internet of Things, sensor networks

## Abstract

This editorial gives an overview of the papers included in the Special Issue on “Security, Privacy, and Trustworthiness of Sensor Networks and Internet of Things” of *Sensors*. The context of the special issue theme is first briefly described. This is then followed by an outline of each paper that provides information on the problem addressed; the proposed solution/approach; and, where relevant, the results of the evaluation of the proposed solution.

## 1. Introduction

The rapid developments in hardware, software, and communication technologies have facilitated the spread of interconnected sensors, actuators and heterogeneous devices. An example is single board computers, which collect and exchange a large amount of data for offering a new class of advanced services characterized by being available anywhere, at any time and for anyone. This ecosystem is widely referred to as the Internet of Things (IoT). In the past years, the number of deployments both for Sensor Networks (SN) and the IoT grew significantly. This continuous and exponential growth is facilitated by investments and research activities originating from industry, academia and governments, while the penetration of these technologies is also driven by the high technology acceptance rates of both consumers and technologists across disciplines. Such networks collect, store, and exchange a large volume of heterogeneous data. Nevertheless, their rapid and widespread deployment, along with their participation in the provisioning of potentially critical services (e.g., safety applications, healthcare, manufacturing), raise numerous issues related to the security, privacy, and trustworthiness of the performed operations and provided services. Accordingly, research into the security and privacy of the IoT and sensor networks is attracting increasing attention from both industry and academia. In line with these efforts, the central theme of this Special Issue is to investigate novel methodologies, theories, technologies, techniques, and solutions for IoT/SN security, trust and privacy. In particular, this Special Issue aims at addressing these topics across multiple abstraction levels, ranging from architectural models, the provisioning of services, protocols and interfaces, as well as specific implementation approaches.

Fifteen papers have been published in this special issue. Brief presentations of these are given in the next section.

## 2. Contributions

In the first paper of the special issue [1], entitled “RESPOnSE—A Framework for Enforcing Risk-Aware Security Policies in Constrained Dynamic Environments”, Michailidou et al. address the problem of enforcing fine-grained access control policies in constrained dynamic networks. They propose the RESPOnSE framework, in which the computational burden is transferred to high-tier nodes, while low-tier nodes apply risk-aware policy enforcement. RESPOnSE builds on a combination of attribute-based access control and role-based access control and is founded on a compensatory multi-criteria decision-making algorithm influenced by TOPSIS. In addition to describing the framework, a use case demonstrating its application in scenarios involving data from a real corporate network is also provided.

In [2], entitled “Data Security and Trading Framework for Smart Grids in Neighborhood Area Networks”, Junior et al. address the issue of end-user privacy in the smart grid. They propose a framework that preserves the secrecy of prosumers’ identities; provides protection against traffic analysis attacks, and hides the amount of bidders and of successful bids in a competitive market for energy trade in a Neighborhood Area Network (NAN). A proprietary communication system is employed, and the Advanced Encryption Standard (AES) 128 bit with Exclusive-OR (XOR) keys is used for encryption. An analysis of the security and privacy features, as well as of the computational cost of the proposed framework indicates that it outperforms the state-of-the-art solutions in terms of privacy protection and trading flexibility in a prosumer-to-prosumer design, especially concerning privacy protection against IP de-anonymization and traffic analysis attacks.

In [3], entitled “A Stepwise and Hybrid Trust Evaluation Scheme for Tactical Wireless Sensor Networks”, Lim et al. consider a tree-based tactical wireless sensor network that includes malicious nodes that perform packet-dropping attacks, and they propose a stepwise and hybrid trust evaluation scheme for locating such nodes. To this end, sensors send a query to the gateway by observing the traffic patterns of their child nodes and, depending on the situation, the gateway detects malicious nodes by choosing between gateway-assisted trust evaluation and gateway-independent trust evaluation. The proposed scheme was implemented and tested on a 30-node network with the OPNET simulator; the results show that the proposed scheme successfully detects malicious nodes performing two types of packet-dropping attacks (gray-hole and smart gray-hole), while exhibiting a higher packet delivery ratio with significantly lower energy consumption than the CENTERA protocol.

In [4], entitled “Information-Aware Secure Routing in Wireless Sensor Networks”, Shi et al. address the problem of secure routing in Wireless Sensor Networks in the presence of malicious nodes. They first establish a network model for multi-hop communications between sensor nodes and the sink node, that describes the behaviors of the sensor nodes. They further describe a method for calculating trust between adjacent nodes, to serve as the basis for the route selection. The trust value (defined as the attack probability), and status (a hybrid metric that combines the residual energy and distance to the sink node) of each relay node in the route are then used to develop a secure routing model based on information-aware sensor nodes. Simulation experiments indicate that the proposed model can maintain a higher delivery ratio than the Reputation-Based Mechanism to Stimulate Cooperation (RBMSC) model and a lower packet loss ratio than the traditional Dijkstra algorithm.

In [5], entitled “A Role-Based Access Control Model in Modbus SCADA Systems. A Centralized Model Approach”, by Figueroa-Lorenzo et al., the security problems of the Modbus protocol which is widely used in Industrial Control Systems (ICS) and Supervisory Control systems and Data Acquisition (SCADA) networks is addressed. Like most such protocols, Modbus was not designed with security in mind, and it is known to suffer from a number of vulnerabilities that can be exploited by several kinds of attacks. The authors propose a new secure version of a role-based access control model (RBAC), in order to authorize both the client on the server, as well as the Modbus frame. The proposed scheme is evaluated by means of both a security analysis and an experimental performance analysis. The results of these evaluations indicate that the proposed scheme is resistant to attacks and its performance in terms of introduced latencies makes its implementation feasible in industrial networks.

In [6], entitled “Mitigating the Impact on Users’ Privacy Caused by over Specifications in the Design of IoT Applications”, by Pérez Fernández and Sindre, the authors address the problem of preserving user privacy in IoT applications in the presence of over-specifications during the system development life cycle. The authors carried out a controlled experiment with students performing an analysis of privacy implications using two different methods. One method aims at reducing the impact of over-specifications through the application of a goal-oriented analysis, whilst the other does not involve a goal-oriented analysis and is used as a control. Initial findings show that conducting a goal-oriented analysis early during design time can have a positive impact over the privacy friendliness of the resulting system.

Paper [7], entitled “False Data Detection for Fog and Internet of Things Networks”, by Fantacci et al. addresses the problem of attack detection in Fog/IoT environments. The authors propose an attack-detection method, named Data Intrusion Detection System (DataIDS), that is based on real-time analysis of physical (sensed) data. As end devices are usually resource-constrained, Fog Computing (FC) is introduced to implement the DataIDS. The DataIDS is able to detect a malicious (or false) data injection by analyzing the data streams acquired by the devices and, also to identify the misbehaving devices. Further, the authors propose an attack tree to model threats and vulnerabilities of Fog/IoT scenarios with heterogeneous devices, and they suggest countermeasure costs. The performance of the proposed DataIDS was examined by implementing a testbed with several devices that measure different physical quantities and by using standard data-gathering protocols. The results of the experimentation indicate that DataIDS has several advantages over existing alternative approaches and that it can be easily implemented in constrained resource devices.

Paper [8], entitled “IoT Security Configurability with Security-by-Contract”, by Giaretta et al. addresses the problem of lack of secure default configurations and sufficient security configurability of IoT devices. The authors envision a future where IoT devices carry behavioral contracts and Fog nodes store network policies, and they propose to combine the security-by-contract (S × C) paradigm with Fog computing to secure IoT devices. Building upon their previous work, they formally define the pillars (security rules, contracts, policies) of the proposal and the notion of consistency for both contracts and policies, which they then use to define several properties useful for the security of IoT systems, such as contract-policy matching and illegal information flow. Then, by means of a running case study, based on a real-world smart home, they illustrate the formally-defined concepts and methods, and they show how the proposed paradigm can be integrated with Fog computing to secure IoT systems successfully.

Paper [9], entitled “An Edge-Fog Secure Self-Authenticable Data Transfer Protocol”, by Venčkauskas et al. addresses the problem of secure communication between constrained-resource devices. In particular, the authors propose a new lightweight, secure self-authenticable transfer protocol (SSATP) for communications between Edge nodes and Fog nodes. The primary target of the proposed protocol is to be used as a secure transport for CoAP (Constrained Application Protocol) in place of the UDP (User Datagram Protocol) and DTLS (Datagram Transport Layer Security) protocols. SSATP uses only symmetric cryptography primitives, and therefore it is easily implementable small devices having low-end processing and memory capabilities. The performance of the proposed protocol has been qualitatively and experimentally evaluated. The results of the experimentation indicate that the SSATP exhibits better transfer performance in applications in which the use of the block-wise mode of CoAP protocol is prevalent. Further, the SSATP provides a better overall data delivery rate in unreliable networks, with up to 10% of total packet loss, and consumes less energy when compared to plain UDP and DTLS working as transport protocols for CoAP in block data transfer mode.

Paper [10], entitled “RPAS Forensic Validation Analysis Towards a Technical Investigation Process: A Case Study of Yuneec Typhoon H”, by Salamh et al. addresses the problem of acquiring and validating forensically sound evidence of the use of Remotely Piloted Aerial Systems (RPAS). The authors propose a technical forensic process consisting of ten technical phases for the analysis of RPAS forensic artifacts, which constitutes a standardized approach to conduct a validated digital forensic analysis of a drone. Using the proposed technical process, the authors analyze drone images using the Computer Forensics Reference Datasets (CFReDS) and present results for the Yuneec Typhoon H aerial vehicle.

Paper [11], entitled “SPS and DPS: Two New Grid-Based Source Location Privacy Protection Schemes in Wireless Sensor Networks”, by Wang et al. addresses the source location privacy protection problem in WSNs and proposes two new grid-based source location privacy protection schemes in WSNs called grid-based single phantom node source location privacy protection scheme (SPS) and grid-based dual phantom node source location privacy protection scheme (DPS). Instead of attempting to determine the phantom node by the source node, as existing schemes do, the authors propose to use a powerful sink node to help the source node to determine the phantom node candidate set (PNCS), from which the source node randomly selects a phantom node acting as a fake source node. The performance of the proposed schemes is evaluated through both theoretical analysis and simulation experiments. The experimental results show that the proposed schemes are more efficient, more secure, and are consuming less total energy than existing alternatives, thus suitable for resource-constrained scenarios.

Paper [12], entitled “A Survey of Context-Aware Access Control Mechanisms for Cloud and Fog Networks: Taxonomy and Open Research Issues”, by Kayes et al. presents a survey of security, privacy and access control research, while highlighting several specific concerns in a wide range of contextual conditions (e.g., spatial, temporal and environmental contexts). The authors present different taxonomies, such as contextual conditions and authorization models, and discuss the existing context-sensitive access control approaches. Further, they propose a new generation of Fog-Based Context-Aware Access Control (FB-CAAC) framework that combines the benefits of the cloud, IoT and context-aware computing. The paper also provides an in-depth analysis of the research challenges in the area that have not been adequately addressed in the literature and leverages this to highlight directions for future work.

Paper [13], entitled “Use Of Smartphones for Ensuring Vulnerable Road User Safety through Path Prediction and Early Warning: An In-Depth Review of Capabilities, Limitations and Their Applications in Cooperative Intelligent Transport Systems”, by Vourgidis et al. looks at the field of cooperative intelligent transport systems and more specifically pedestrians to vehicles. Pedestrians to vehicles is a type of cooperative intelligent transport system, within the group of early warning collision/safety system. The authors review the research and applications within the field of pedestrians to vehicles’ cooperative transport systems by leveraging the information coming from road users’ smartphones. Further, they review the literature on outdoor localization and next-step/movement prediction of road users via smartphones. They then identify areas of possible improvement, and they address future research objectives and methodologies that could support the identified areas.

Paper [14], entitled “Survey on Revocation in Ciphertext-Policy Attribute-Based Encryption”, by Al-Dahhan et al. surveys revocation issues and lack of managing a wide range of attributes when Ciphertext-Policy Attribute-Based Encryption (CP-ABE) is used in Internet of Things (IoT) applications. The authors review existing single- and multi-authority CP-ABE schemes, focusing on their ability to address the revocation issues, and the techniques used to manage revocation. The survey results are then used to propose challenging areas of relevant research.

The last paper in the special issue [15], entitled “Detecting IoT Devices and How They Put Large Heterogeneous Networks at Security Risk”, by Agarwal et al. proposes a novel method to detect IoT devices and identify the manufacturer, device model, and the firmware version currently running on the device by using the page source from the web user interface. The authors also developed two software tools that support the application of the method. The approach was evaluated by performing automatic scans of the large-scale network at the European Organization for Nuclear Research (CERN). The developed tools identified 233 IoT devices that fell into eleven distinct device categories and included 49 device models manufactured by 26 vendors from across the world, thus providing evidence that the approach is effective on a large-scale network with a larger dataset than those used in similar studies.
